# Evaluation of the tolerance and forage quality of different ecotypes of seashore paspalum

**DOI:** 10.3389/fpls.2022.944894

**Published:** 2022-09-29

**Authors:** Kai Jiang, Zhimin Yang, Juan Sun, Huancheng Liu, Shenmiao Chen, Yongzhuo Zhao, Wangdan Xiong, Wenjie Lu, Zeng-Yu Wang, Xueli Wu

**Affiliations:** ^1^ College of Grassland Science, Qingdao Agricultural University, Qingdao, China; ^2^ College of Grassland Science, Nanjing Agricultural University, Nanjing, China

**Keywords:** seashore paspalum, forage quality, crude protein contents, salinity, low temperature

## Abstract

Seashore paspalum is a halophytic, warm-season grass with wide applications. It is noted for its superior salt tolerance in saline environments; however, the nutritive value of seashore paspalum and the effect of salinity remains to be determined. Therefore, this study aimed to evaluate the relationship between agronomic traits and forage quality and identified the effects of short-term high-salt stress (1 week, 700 mM NaCl) on the growth and forage nutritive value of 16 ecotypes of seashore paspalum. The salt and cold tolerances of the seashore paspalum ecotypes were assessed based on the survival rate following long-term high-salt stress (7 weeks, 700 mM NaCl) and exposure to natural low temperature stress. There were significant genetic (ecotype-specific) effects on plant height, leaf–stem ratio, and survival rate of seashore paspalum following salt or low temperature stress. Plant height was significantly negatively correlated with the leaf–stem ratio (*r* = −0.63, *P*<0.01), but the heights and leaf–stem ratios were not significantly correlated with the fresh weight (FW) and dry weight (DW) of the shoots. High salinity decreased the FW and DW of the shoots by 50.6% and 23.6%, respectively, on average. Seashore paspalum exhibited outstanding salt tolerance and forage quality at high salinity. The survival rate of the different ecotypes of seashore paspalum varied from 6.5% to 49.0% following treatment with 700 mM NaCl for 7 weeks. The crude protein (CP) content of the control and treatment groups (700 mM NaCl) was 17.4% and 19.3%, respectively, of the DW on average, and the CP content of most ecotypes was not significantly influenced by high salinity. The average ether extract (EE) content ranged from 4.6% to 4.4% of the DW under control and saline conditions, respectively, indicating that the influence was not significant. The neutral detergent fiber (NDF) and acid detergent fiber (ADF) contents of the control group were 57.4% and 29.8%, respectively, of the DW on average. Salt stress reduced the content of NDF and ADF to 50.2% and 25.9%, respectively, of the DW on average. Altogether, the results demonstrated that stress did not have any significant effects on the CP and EE content of most ecotypes, but reduced the NDF and ADF content and improved relative feed value (RFV). The results obtained herein support the notion that seashore paspalum is a good candidate for improving the forage potential of saline soils and can provide useful guidelines for livestock producers.

## Introduction

Soil salinization is a major environmental concern affecting global agricultural production and the ecological environment (Anderson et al., 2015; Yin et al., 2019). Salinization affects approximately 7% of the land surface worldwide, threatens nearly 20% of global irrigated land, and the proportion of saline soils is increasing (Li et al., 2014; Gangwar et al., 2020; Mora et al., 2020). The total area of saline soil in China is approximately 3.6 × 10^7^ ha, which accounts for 4.88% of the total available land area of the country (Dong et al., 2019; Zhang et al., 2022). Saline-alkali lands are one of the limiting factors for crop production and ecological construction. Soil salinity and low temperature are the major environmental constraints that severely affect the growth and productivity of crop plants (Robinson et al., 2004; Moustafa et al., 2021). Plants growing under salt-stressed conditions are challenged by ionic imbalance, impaired photosynthesis, osmotic stress, and nutrient deficiency, which seriously inhibit plant growth and quality (Huang et al., 2015; Munns and Gilliham, 2015; Oney-Birol, 2019). However, these lands can be used as an important reserve land resource (Munir et al., 2022). Soil engineering and agronomic solutions for alleviating soil salinity are ineffective to date, and growing salinity-tolerant plants can improve the utilization rate of saline-alkali lands (Gu et al., 2019). The protection of arable lands and the development of saline-alkali lands will be important measures for ensuring food security and economic and population growth. The development of animal husbandry in saline areas is an important measure for multifunctional ecological agriculture. But, the supply of forage is an important limiting factor for the development of animal husbandry in saline-alkali areas. Owing to poor salt tolerance, the yield and quality of traditional forage plants, including alfalfa (*Medicago sativa* L.) (Campanelli et al., 2013; Badran et al., 2015; Monirifar et al., 2020), sweet sorghum (*Sorghum bicolor* L. Moench) (Yang et al., 2020), and forage kochia (*Bassia prostrata* L.) (Waldron et al., 2020), are affected by salt stress. However, halophytes can grow well at high salt concentrations (200 mM NaCl and higher) as they possess highly special mechanisms of salt resistance (Yuan et al., 2019; Shoukat et al., 2020). Halophytic forage plants or grasses can provide a basis for the development of halophyte-based agricultural practices with the aim of increasing the security and yield of agricultural and livestock products (Norman et al., 2019; Munir et al., 2022).

Seashore paspalum (*Paspalum vaginatum* O. Swartz) is a halophytic, warm-season grass that is commonly grown on athletic fields, golf courses, and landscape areas. Seashore paspalum grows extensively in saline regions of the landscape and has an immense potential to be used under harsh and stressful environmental conditions (Pessarakli, 2018). The plant is favored owing to its high tolerance to salt, drought, water logging, low soil pH, and adaptation to conditions of low irradiance and weak shade (Pompeiano et al., 2016; Karimi et al., 2018). Evaluation of the salt tolerance of seashore paspalum and other species of grass indicates the superior salt tolerance of seashore paspalum (Marcum and Murdoch, 1994). Seashore paspalum has better ion regulation abilities that are mediated *via* its ability to maintain a high concentration of K^+^ in shoots and the reduced transfer of Na^+^ from roots to shoots, which leads to the maintenance of a high K^+^/Na^+^ ratio. Therefore, seashore paspalum is capable of evading ion-specific damage resulting from salt stress (Schiavon et al., 2012; Wu et al., 2018). Moreover, the plant has an enhanced Ca^2+^ signaling transduction pathway resulting from Na^+^ accumulation, which aids in maintaining the activities of the major antioxidant enzymes (Wu et al., 2020).

Seashore paspalum has the second largest number of cultivars in the *Paspalum* genus, of which the majority of cultivars have been developed in the USA, particularly at the University of Georgia (Acuña et al., 2019). Molecular markers are used for the effective identification of phenotypic variations and genetic differences among ecotypes during the collection and evaluation of germplasm resources. Microsatellite sequence markers are usually species-specific; however, few such markers are available for *P*. *vaginatum* (Cidade et al., 2013). Using 43 simple sequence repeat (SSR) markers, a previous study determined the relationship between *P. vaginatum* and *P. distichum*, and determined their genetic diversity and ploidy levels (Eudy et al., 2017). Previous studies have analyzed the diversity and variations among different ecotypes of seashore paspalum using random amplified polymorphic DNA (RAPD) markers (Liu et al., 1994), amplified fragment length polymorphisms (AFLPs) (Chen et al., 2005), and SSR markers (Wang et al., 2006). A previous study developed several thousand single-nucleotide polymorphism (SNP) markers that segregated in an F1 population, generated from a cross between ecotypes 509022 and HI33 of seashore paspalum. The markers were subsequently used to construct the first genetic maps for this plant (Qi et al., 2019). Although seashore paspalum has been utilized for almost a hundred years and numerous cultivars have been developed, few genetic resources are available for this plant to date (Qi et al., 2019). There is a major difference between the salt- and cold-tolerant varieties of seashore paspalum (Cardona et al., 1997). ‘SI92’ and ‘SI93-1’ are salt-tolerant varieties, while ‘SI95’ and ‘Adalayd’ have the worst salt tolerance (Lee et al., 2004). Several recent studies have evaluated stress (salt, drought, etc.) tolerance (Karimi et al., 2018; Tran et al., 2018) and determined the underlying regulatory mechanism in different varieties of seashore paspalum (Cyril et al., 2002; Pompeiano et al., 2016; Serena et al., 2018; Wu et al., 2018). Seashore paspalum has remarkable salt tolerance properties; but its low tolerance to cold environments seriously affects its application in regions with low temperatures. However, there is a scarcity of studies evaluating the nutritive value of seashore paspalum during salt response.

The cultivation of halophytes for agriculture and animal husbandry development needs is recommended due to their ability to grow in saline-degraded lands, which provides environmental and economic benefits to society (Himabindu et al., 2016). However, most studies on salt-tolerant forage plants have emphasized evaluating biomass growth and physiological mechanisms, whereas few studies have focused on determining the effect of salinity on the forage quality of halophyte species. Therefore, further studies are necessary to evaluate the effect of salinity on the forage quality of halophytes (Norman et al., 2013; Waldron et al., 2020). Seashore paspalum is a natural inhabitant of saline soils and has numerous potential applications for sustaining excellent forage productivity, which can improve forage yield and, in particular, improve the usage rate of saline lands. Seashore paspalum is noted for its high salt tolerance ability. However, the forage quality and effect of salinity on its nutritive value remain to be determined. Therefore, this study determined the effect of salinity on the forage quality of seashore paspalum. In this study, we evaluated the low temperature and high salt tolerance of seashore paspalum, determined the relationship between biomass and photosynthesis, and investigated the forage nutritive value of seashore paspalum under salinity conditions. In this study, cold- and salt-tolerant ecotypes of seashore paspalum with high biological yield and high nutritional quality were selected to provide a basis for agriculture and animal husbandry development needs in saline lands.

## Materials and methods

### Plant growth and treatments

**Figure 1:**
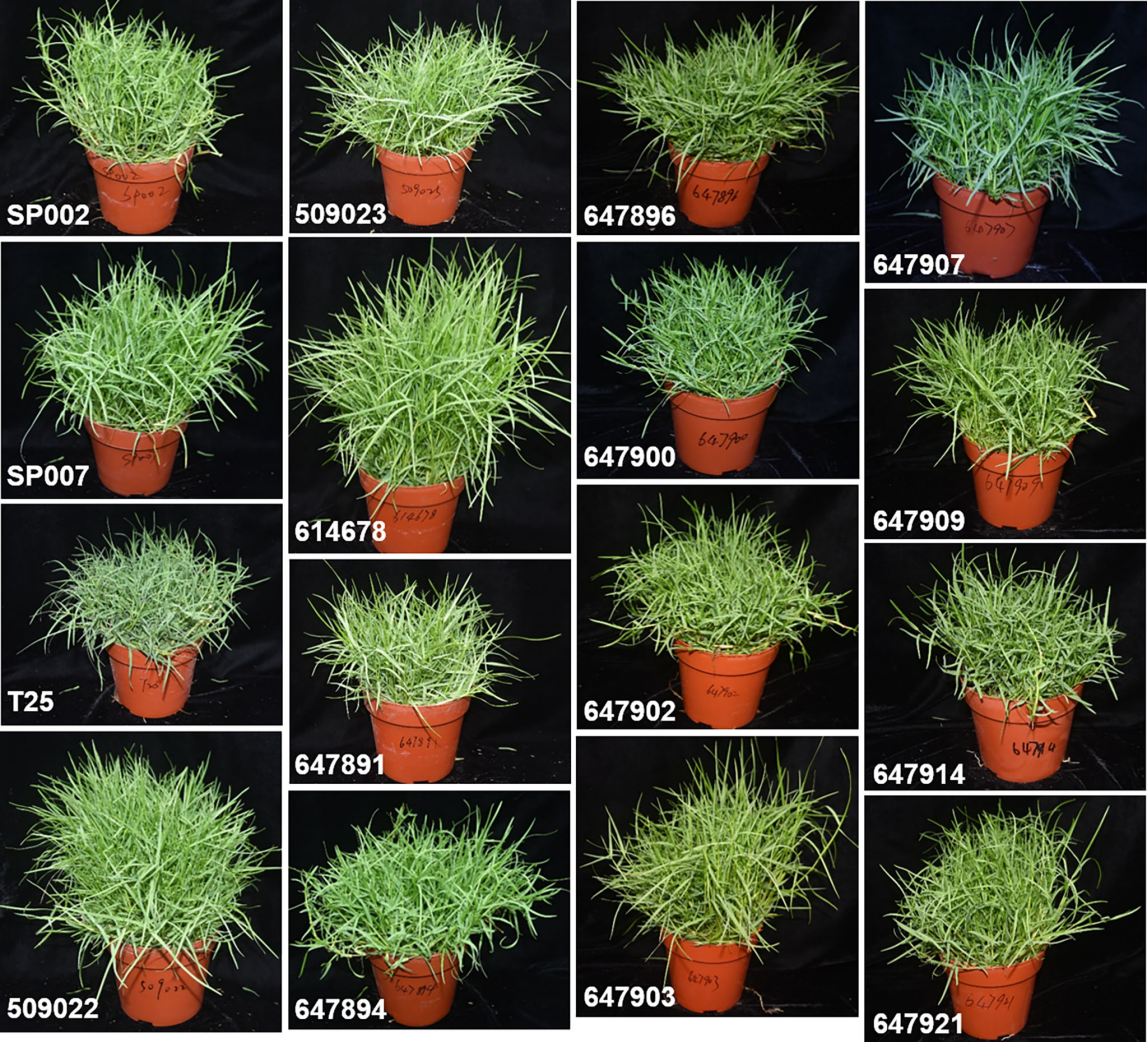
Characterization of 16 seashore paspalum ecotypes under normal condition for 40 d.

In this study, 16 ecotypes of seashore paspalum were collected from coastal environments and golf courses (**Table 1**). The 16 ecotypes showed genetic divergence in terms of various characteristics, including plant height, length and width of leaves, tillers/plant, leaf color, spreading growth pattern, plant density, and tolerance. For instance, we observed that 509022 and 614678 plants were taller, had longer leaves, and faster growth rates than the other ecotypes. Contrarily, T25 plants were short, and their leaves were short and soft. The leaf color of 647907 and T25 plants was darker than that of the other ecotypes (**Figure 1**). The 16 seashore paspalum ecotypes were planted in soil containing a mixture of peat and perlite (3:1) in isolated plastic pots. Each ecotype was multiplied by vegetative propagation in nine uniform plastic pots, and 50 stem segments were planted per pot. The plants were fully irrigated and grown in a greenhouse under natural light. Throughout the experiment, the day and night temperatures were maintained at 30°C and 25°C, respectively, and the relative humidity was maintained at 50% during the day and 70% during the night.

**Table 1:** The sources of 16 seashore paspalum ecotypes.

Entry	Origin	Entry	Origin
SP002	China	PI 647896	United States of America
SP007	China	PI 647900	United States of America
T25	China	PI 647902	United States of America
PI 509022	United States of America	PI 647903	United States of America
PI 509023	United States of America	PI 647907	United States of America
PI 614678	United States of America	PI 647909	United States of America
PI 647891	United States of America	PI 647914	United States of America
PI 647894	United States of America	PI 647921	United States of America

The stress treatments were initiated when the plants had been growing in the greenhouse for 8 weeks, and various indicators were measured. For each ecotype, three pots of plants were designated as the control, which were routinely maintained under normal growth conditions. For measuring plant height, three of the tallest tillers were selected randomly per ecotype, and the height was measured in cm from the level of the soil to the tip of the tallest leaf with a ruler. The tillers were manually separated into leaves and stems, and subsequently oven dried at 65°C for 96 h to determine the DW for estimation of the leaf–stem ratio. The second fully developed leaves from the top were used for measuring the chlorophyll content and determining the photosynthesis-related parameters in three replicates. For measurement of the chlorophyll content, the leaves (0.1 g) were fully extracted for 48 h in 10 ml of 95% ethanol (v/v) in the dark. The absorbance at 645 nm and 663 nm was determined using a spectrophotometer as previously described (Wu et al., 2018). The net photosynthetic rate (Pn), intercellular CO_2_ concentration (Ci), transpiration rate (Tr), and stomatal conductance (Gs) were measured using the LI-6400-40 portable photosynthesis system at 25°C and 400 ppm CO_2_.

For salinity treatments, three pots of plants per ecotype were irrigated with 400 mM NaCl solution for 3 days (d) and then with 700 mM NaCl for 1 week. All the tillers in the pots were clipped to a height of 5 cm from the soil surface. The relative growth of the plants was calculated under control conditions using the formula: fresh weight (FW)/duration of growth (d). The FW was recorded separately under control and saline conditions. For determination of the DW, herbage mass, and forage nutritive value, the harvested samples were dried in a forced air oven at 105°C for 30 min and then at 65°C until a constant weight was reached. The water content (WC) and dry-fresh weight ratio (DFR) of the plants were measured under control and salinity treatment conditions. The WC was calculated using the following formula: WC = [(FW − DW)/FW] × 100% (Gitau et al., 2017). The DFR was calculated using the formula: DFR = FW/DW. The dried shoot samples were ground to a particle size of 1 mm using an automatic grinding machine for analysis of forage nutritive value. The analyses were performed in triplicate for each sample.

### Assessment of forage nutritive value

The forage nutritive value was determined in triplicate based on the DW. The total concentration of N and C was determined by dry combustion using a Vario EL cube elemental analyzer (Elementar, Germany), and the crude protein (CP) content (% DW) was calculated as 6.25 × N concentration. The ether extract (EE) content was determined according to the Chinese Standard GB/T 6435-1986. Briefly, the samples (1.0 g) were placed in individual test bags and sealed, following which they were dried at 65°C for 3 h. Petroleum ether was added to an ANKOM XT15 automatic fat analyzer (ANKOM Technology, USA) for extraction and determination. The crude fat content was calculated from the changes in weight (Ji and Wang, 2002). The contents of the neutral detergent fiber (NDF; % DW) and acid detergent fiber (ADF; % DW) were sequentially determined using an ANKOM A2000i automatic fiber analyzer (ANKOM Technology, USA). Briefly, the samples (0.5 g) were placed in dedicated filter bags (F57 or F58, ANKOM Technology, USA) and sealed. The NDF and ADF contents were measured using the filter bag technology, according to the detailed instructions in the ANKOM A2000i manual. The digestible dry matter (DDM; % DW), dry-matter intake (DMI; % DW), and the relative feed value (RFV; % DW) were calculated on a DW basis using the following formulas (Kim and Anderson, 2015):


DDM = 88.9 – (0.779 × ADF)

DMI = 120 / NDF

RFV = (DDM × DMI) / 1.29

### Assessment of salt and cold tolerance

The salt and cold tolerance of the plants was also evaluated from the survival rate. The NaCl concentration was increased to 700 mM for 7 weeks, and the samples were photographed after harvesting. The survival rate was subsequently calculated after salt treatment by watering for 7 d to wash out the salt. The survival rate (%) was calculated as: (number of plants that survived after watering/the total number of plants) × 100.

To evaluate cold tolerance, three pots of plants were removed per ecotype from the greenhouse and maintained at natural low temperatures for 9 d and 15 d at Qingdao Agricultural University, situated in southeast Shandong on the northern shore of the Huanghai Sea (37° 09’ N, 119° 30’ E). The area has a temperate monsoon climate, and the average annual temperature of this area is 12.7°C. The temperature during cold treatment was recorded. The survival rate (%) was calculated as: (number of plants that survived after returning to 25°C/total number of plants) × 100.

### Data analyses

Data from three replicates were analyzed by one-way analysis of variance (ANOVA). The results are presented as the mean ± standard error of biological replications. The means were separated using Duncan’s multiple range test (*P*<0.05). Statistical analyses were performed using the Statistical Package for the Social Sciences (SPSS; version 17.0). The correlations among the features were estimated with Pearson’s correlation coefficient test, performed using the appropriate method in SPSS.

## Results

### Analysis of agronomic traits and photosynthesis-related parameters

**Figure 2:**
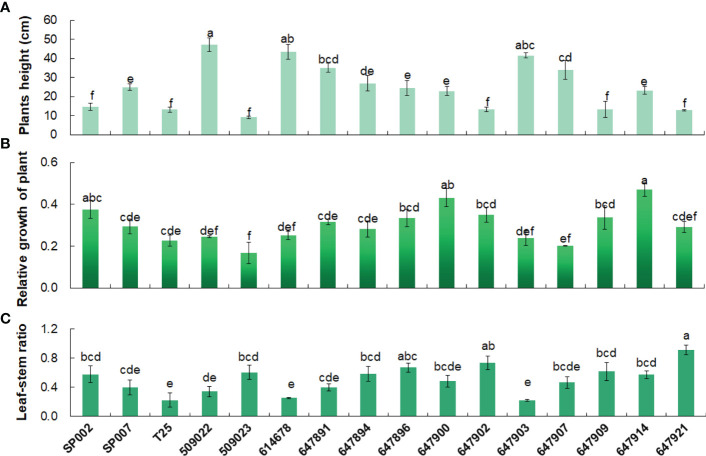
Agronomic traits analysis of 16 seashore paspalum ecotypes. Plants height **(A)**, Relative growth of plant **(B)** and Leaf–stem ratio **(C)** of 16 seashore paspalum ecotypes were measured when plants grown in the greenhouse for 8 weeks. Means of three replicates and standard errors are presented; the same letter above the column indicates no significant difference at *P*<0.05.

Analyses of the agronomic traits revealed significant genetic (ecotype-specific) differences in plant height and leaf–stem ratio. Plant height is one of the important traits that affect the yield, and in this study, the height of the plants was significantly negatively correlated with the leaf–stem ratio (*r* = −0.63, *P*< 0.01) **(** **Table 2** **)**. For instance, we observed that 509022 and 614678 plants were significantly taller and had significantly lower leaf–stem ratios than those of 647902 and 647921 plants. The heights of the plants in the different ecotypes ranged from 47.10 cm for 509022 plants to 9.10 cm for 509023 plants. The 509022, 614678, and 647903 plants were significantly taller than SP002, SP007, T25, 509023, 647894, 647896, 647900, 647902, 647909, 647914, and 647921 plants. Leaf proportion has important effects on forage nutritive value, and it was found to be affected by the ecotype of the plants. The leaf–stem ratio of 647921 plants was significantly higher than that of 647903 plants (0.92 vs. 0.22, *P*< 0.05). The 647921 plants had a more horizontal and prostrate growth pattern compared to that of 647903 plants, and the shorter canopy height of 647921 resulted in an increased leaf proportion and decreased stem elongation **(** **Figures 2A, C** **)**. The relative growth of the 16 ecotypes of seashore paspalum ranged from 0.17 to 0.47 **(** **Figure 2B** **)** and was not significantly correlated with the quantitative traits in **Table 2**, according to Pearson’s correlation coefficient test.

**Table 2:** Pearson’s correlation coefficients between pairs of traits.

	Relative growth of plant	Leaf–stem ratio	Plants height	Chlorophyll contents	Pn	Ci	Tr	Gs
Relative growth of plant	–	0.34	−0.22	−0.21	0.03	−0.06	−0.02	−0.08
Leaf–stem ratio		–	−0.63**	0.03	0.21	−0.46	−0.18	−0.18
Plants height			–	−0.11	−0.07	0.13	−0.18	−0.17
Chlorophyll contents				–	0.41	−0.42	0.29	0.19
Pn					–	−0.69**	0.60*	0.62*
Ci						–	0.03	0.06
Tr							–	0.97**
Gs								–

*P<0.05, **P<0.01 represent significant values that been bold in tables.

Pn, net photosynthetic rate; Ci, intercellular CO_2_ concentration; Tr, transpiration rate; Gs, stomatal conductance.

**Figure 3:**
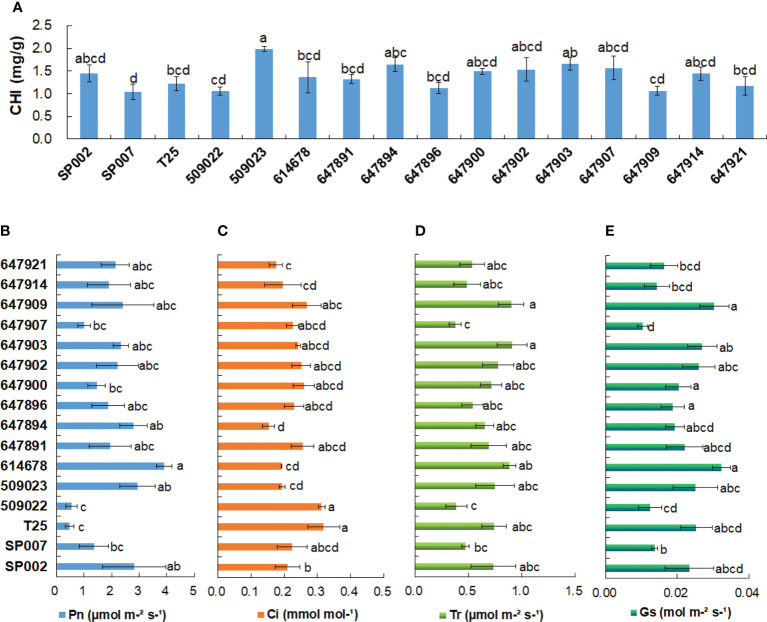
Chlorophyll contents and photosynthesis-related parameters analysis of 16 seashore paspalum ecotypes. Chlorophyll contents **(A)**, net photosynthetic rate (Pn) **(B)** and intercellular CO_2_ concentration (Ci) **(C)**, transpiration rate (Tr) **(D)**, and stomatal conductance (Gs) **(E)** were measured by LI-6400-40 portable photosynthesis system when 16 seashore paspalum ecotypes grown in the greenhouse for 8 weeks. Means of three replicates and standard errors are presented; the same letter above the column indicates no significant difference at *P*<0.05.

The chlorophyll content of the different ecotypes ranged from 1.04 mg/g for SP007 plants to 1.98 mg/g for 509023 plants. The 509023 plants had the highest chlorophyll content, which was significantly higher than that of SP007, T25, 509022, 614678, 647891, 647896, 647909, and 647921 plants **(** **Figure 3A** **)**. The results of Pearson’s correlation coefficient test revealed that the correlation between the chlorophyll content and Pn was not significant (*r* = 0.41, *P >*0.05) (**Table 2**). However, chlorophyll is an important pigment that is necessary for photosynthesis in plants, and the chlorophyll content can reflect the degree of photosynthesis. For instance, the chlorophyll content and Pn of 509022 plants were significantly lower than those of 509023 plants, and a similar trend was observed in the chlorophyll content and Pn **(** **Figures 3A, B** **)**. Photosynthesis provides energy for plant growth and development and is an important criterion for evaluating plant productivity. The photosynthetic parameters that represent photosynthetic ability and efficiency were monitored under normal conditions. It was observed that the Pn varied between 0.47 and 3.90 µmol m^−^²s^−^¹, and there were minor differences in the Pn among the majority of ecotypes studied herein. For instance, we observed that the Pn of T25 and 509022 plants was significantly lower than that of SP002, 509023, and 614678 plants **(** **Figure 3B** **)**. The Pn was strongly negatively correlated with the Ci **(** **Figure 3C** **)** (*r* = −0.69, *P*<0.01) but positively correlated with the Tr **(** **Figure 3D** **)** (*r* = 0.60, *P*<0.05) and Gs **(** **Figure 3E** **)** (*r* = 0.62, *P*<0.05) (**Table 2**).

### Effect of salt stress on shoot biomass parameters

**Figure 4:**
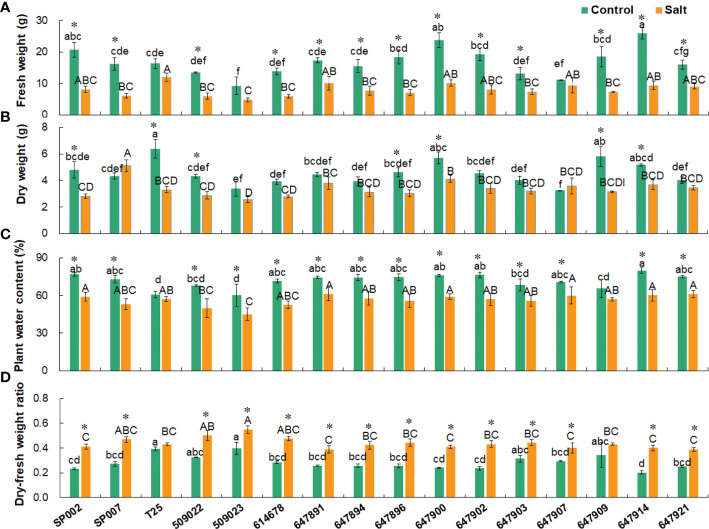
Effect of salt stress on shoot biomass parameters of 16 seashore paspalum ecotypes. Shoots were sampled for measurement of fresh weight **(A)**, dry weight **(B)**, water content **(C)**, and dry- fresh weight ratio **(D)** before treatment (control) and after NaCl treatment for 10 d (salt). Means of three independent experiments and standard errors are presented; the same letter above the column indicates no significant difference at *P*<0.05. Lowercase letters and uppercase letters respectively indicate significant comparisons among different ecotypes in the control group and treatment group. * indicates significant difference at *P*<0.05 by T-test between salt treatment and control for 16 ecotypes.

Salt stress significantly reduced the FW of the plants, with the exception of T25, 509023, and 647907 plants. The FW of the plants was 16.53 g on average under control conditions and 7.99 g on average following salt treatment **(** **Figure 4A** **)**. The FWs of the plants were strongly positively correlated with the WC but negatively correlated with the DFR. The DW was strongly correlated with the FW only under control conditions (*r* = 0.674, *P*< 0.01) (**Table 3**). For instance, the FW (25.89 g) and WC (79.85%) were high in 647914 plants, but the DFR was low (0.20) in all the ecotypes under control conditions **(** **Figures 4A, C, D** **)**. The DW of the majority of ecotypes decreased under salt treatment, with the exception of SP007 and 647907 plants, in which the DWs were not significantly different but increased only slightly **(** **Figure 4B** **)**. The results of Pearson’s correlation coefficient test revealed that the DFRs of plants under control and saline conditions were highly significantly correlated (*r* = 0.625, *P*<0.01) (**Table 3**). The DFR significantly increased in most ecotypes following salt treatment, but there was no significant difference in the DFRs of T25 and 647909 plants **(** **Figure 4D** **)**. This was primarily attributed to the reduction in FW caused by water loss from the plants under saline conditions.

**Table 3:** Pearson’s correlation coefficients between pairs of traits.

	FW control	DW control	W Ccontrol	DFR control	F Wsalt	DW salt	WC salt	DFR salt
FW control	–	0.67**	0.67**	−0.67**	0.46	0.33	0.57*	−0.57*
DW control		–	−0.04	0.04	0.55*	0.17	0.31	−0.31
WC control			–	−0.99**	0.19	0.35	0.60*	-0.60*
DFR control				–	−0.21	−0.37	−0.62**	0.62**
FW salt					–	0.29	0.79**	−0.78**
DW salt						–	0.34	−0.33
WC salt							–	−0.99**
DFR salt								–

*P<0.05, **P<0.01 represent significant values that been bold in tables.

FW, fresh weight; DW, dry weight; WC, water content; DFR, dry-fresh weight ratio.

### Effect of salt stress on forage nutritive value

**Figure 5:**
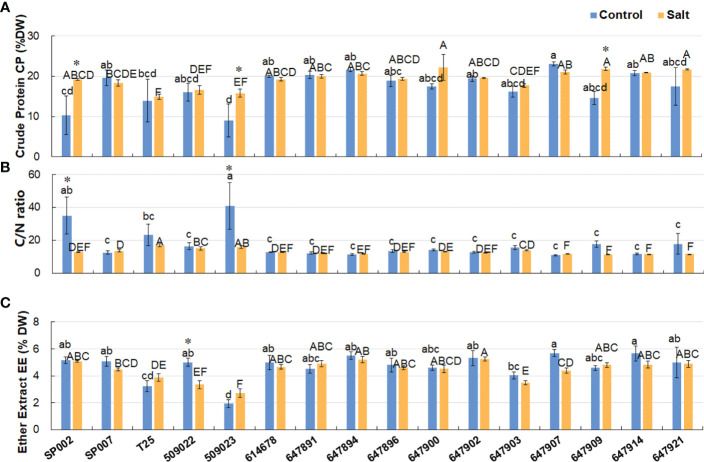
Effect of salt stress on crude protein, C/N ratio, and ether extract of 16 seashore paspalum ecotypes. The Crude Protein CP **(A)**, C/N ratio **(B)**, and Ether Extract EE **(C)** of shoots were measured before treatment (control) and after NaCl treatment for 10 d (salt). Means of three independent experiments and standard errors are presented; the same letter above the column indicates no significant difference at *P*<0.05. Lowercase letters and uppercase letters respectively indicate significant comparisons among different ecotypes in the control group and treatment group. * indicates significant difference at *P*<0.05 by T-test between salt treatment and control for 16 ecotypes.

The CP content of the different ecotypes ranged from 9.04% to 23.04% of the DW, and the average CP content was 17.42% of the DW under control conditions. The results demonstrated that salt stress did not have any significant effects on the CP content of most varieties (19.31% of DW on average), but significantly increased the CP content of SP002, 509023, and 647909 plants **(** **Figure 5A** **)**. Additionally, the C/N ratio of the majority of ecotypes was not significantly affected by salinity (17.34 and 13.08 on average under control and saline conditions, respectively), with the exception of SP002 and 509023 plants. The C/N ratio of SP002 and 509023 plants decreased significantly from 34.91 and 40.88, respectively, under control conditions, to 12.71 and 15.90, respectively, under saline conditions **(** **Figure 5B** **)**. The EE and CP contents of 647907 plants were high among the 16 ecotypes, being 5.70% and 23.04%, respectively, of the DW, while those of 509023 plants were low, being 1.93% and 9.04%, respectively, of the DW under control conditions **(** **Figures 5A, C** **)**. The average EE content ranged from 4.69% to 4.44% of the DW under control and saline conditions, respectively, indicating that the saline environment had no significant influence on the EE content of the plants, with the exception of 509022 plants, in which the EE content decreased significantly under saline conditions **(** **Figure 5C** **)**.

**Figure 6:**
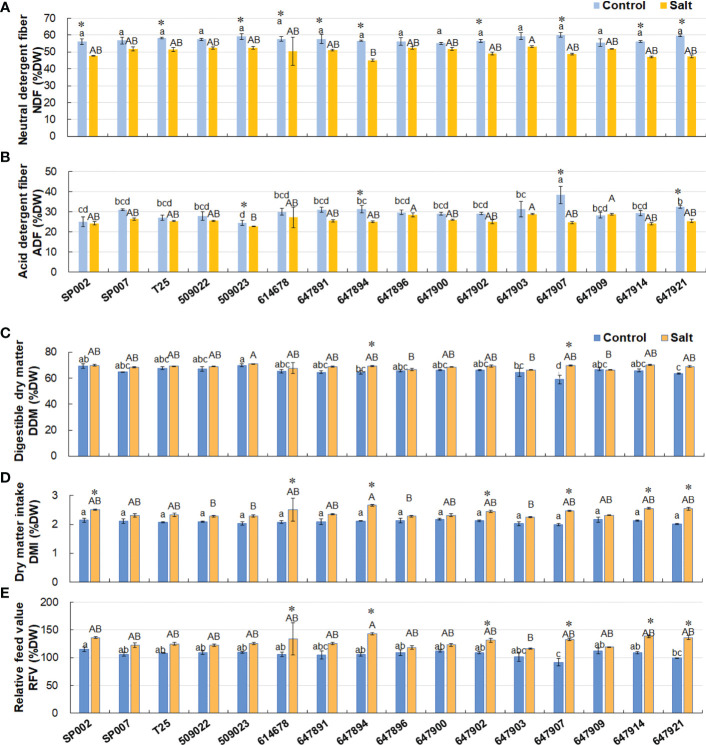
Effect of salt stress on forage nutritive value of 16 seashore paspalum ecotypes. The neutral detergent fiber NDF (% DW) **(A)** and acid detergent fiber ADF (% DW) **(B)** were measured before treatment (control) and after NaCl treatment for 10 d (salt); then digestible dry matter, DDM (% DW) **(C)**, dry-matter intake, DMI (% DW) **(D)**, and relative feed value (RFV) **(E)** were determined. Means of three independent experiments and standard errors are presented; the same letter above the column indicates no significant difference at *P*<0.05. Lowercase letters and uppercase letters respectively indicate significant comparisons among different ecotypes in the control group and treatment group. * indicates significant difference at *P*<0.05 by T-test between salt treatment and control for 16 ecotypes.

All the ecotypes had a higher content of NDF (57.4% of DW on average) under control conditions. Salt stress significantly decreased the NDF content to 50.2% of DW on average, with the exception of SP007, 509022, 647896, 647900, 647903, and 647909 plants **(** **Figure 6A** **)**. There were no significant differences in the ADF content between control and saline conditions, with the exception of 509023, 647894, 647907, and 647921 plants. However, as an advanced forage breeding ecotype, 647907 had a high forage nutritive value, indicated by the high values of ADF, NDF, CP, and EE **(** **Figures 5A, C**, **6A, B** **)**. The forage quality parameters, including DDM, DMI, and RFV, calculated from the content of ADF and NDF, increased in all ecotypes under saline conditions (68.7%, 2.4%, and 128.2%, respectively, on average). There was a small difference in the DDM, DMI, and RFV of the majority of ecotypes, which were 65.7%, 2.1%, and 106.7%, respectively, on average, under control conditions. The DDM ranged from 58.9% (647907) to 69.8% (509023), while the DMI ranged from 2.0% (647907) to 2.18% (647900), and the RFV varied between 91.5% (647907) and 115.0% (SP002) across the 16 ecotypes **(** **Figures 6C–E** **)**.

### Analysis of salt and cold tolerance

**Figure 7:**
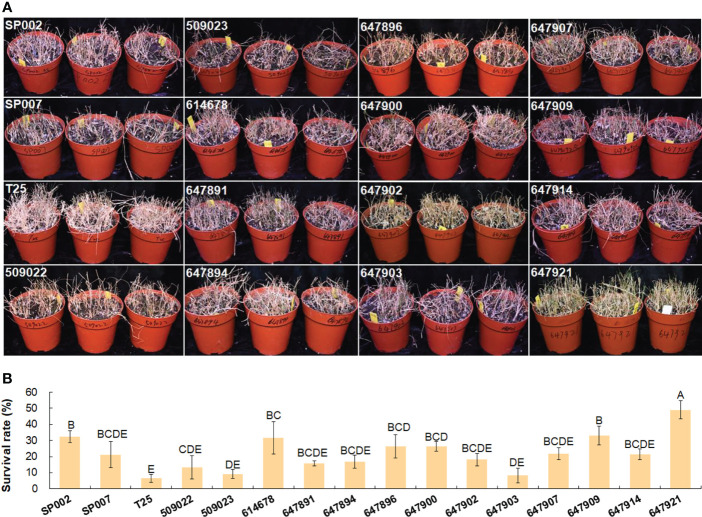
Analysis of salt tolerance in 16 seashore paspalum ecotypes. Photographs were taken when NaCl concentration was increased to 700 mM for 7 weeks **(A)**; then survival rates were calculated after salt treatment by watering to wash salt for 7 d **(B)**. Means of three replicates and standard errors are presented; the same letter above the column indicates no significant difference at *P*<0.05.

The effect of short-term high-salt stress (1 week, 700 mM NaCl) on the growth and forage nutritive value of seashore paspalum was evaluated in this study. The salt tolerance of the seashore paspalum ecotypes was subsequently evaluated by determining the survival rate after long-term high-salt stress (7 weeks, 700 mM NaCl). The evaluation of survival rate is a fundamental strategy for selecting grasses that can be grown in areas with saline land and low temperature conditions. The survival rates after salt and low temperature stress were significantly influenced by the ecotype of the plants. The survival rate of the 16 seashore paspalum ecotypes varied from 49.0% for 647921 plants to 6.5% for T25 plants under long-term high-salt stress **(** **Figures 7A, B** **)**.

**Figure 8:**
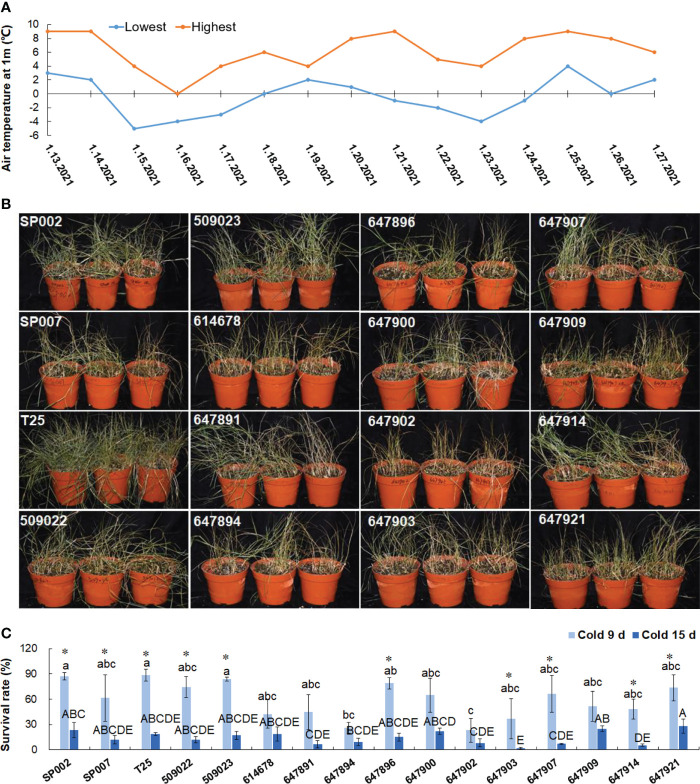
Analysis of low temperature tolerance in 16 seashore paspalum ecotypes. Temperature change diagram of plants treated with low temperature for 15 d **(A)**. Plants were photographed after 9 d under low temperature treatment **(B)**. Survival rates were respectively documented for 9 and 15 d **(C)**. Means of three replicates and standard errors are presented; the same letter above the column indicates no significant difference at *P*<0.05. Lowercase letters and uppercase letters respectively indicate significant comparisons among different ecotypes in cold 9 d and cold 15 d. * indicates significant difference at *P*<0.05 by T-test between cold 9 d treatment and cold 15 d treatment for 16 ecotypes.

To evaluate cold tolerance, the plants were subjected to short-term (9 d) and long-term (15 d) natural low-temperature treatments, where the temperature ranged from −5 °C to 9 °C **(** **Figure 8A** **)**. The survival rate at low temperatures was influenced by the ecotypes of the plants, and ranged from 23.3% (647902) to 88.6% (T25) under short-term low temperature stress, but the effect of the ecotype was not a significant influencing factor for most plants (59.5% on average), with the exception of 647897 and 647902 plants under short-term low temperature stress. Long-term low temperature stress significantly decreased the survival rate of the plants by 14.4% on average, with the exception of 614678, 647891, 647894, 647900, 647902, and 647909 plants **(** **Figures 8B, C** **)**. The survival rate of the plants during short-term and long-term natural low-temperature treatments was significantly positively correlated (*r* = 0.534, *P*<0.05).

## Discussion

Freshwater resources and agricultural prime lands should be allocated for growing food crops for the increasing human population. However, saline lands can be used for the cultivation of halophytes for ecological benefits. Halophytes are expected to have an increasingly important role as genetic resources for the re-vegetation of saline lands (Flowers and Colmer, 2015). Seashore paspalum can produce feed for livestock and grow on poor-quality soils that are unfit for growing crops or for traditional pasture production and can serve as a forage grass for value-added development of saline lands. An early study evaluating the yield and quality of seashore paspalum under saline conditions demonstrated that seashore paspalum has the highest ash content (15.5%) and DW yield, which are almost unaffected by soil salinity. The study revealed that seashore paspalum could show promise in forage production under conditions of high salinity (Pasternak et al., 1993).

### Relationship between agronomic traits and forage quality

Compared with cold-season grasses, perennial warm-season grasses typically have the potential to produce higher biomass yields (Anderson et al., 2015). In a previous study, the forage yield of meadow bromegrass (*Bromus riparius* Rehm) was correlated with plant height and branching ability (de Araujo and Coulman, 2002). In the present study, we selected the warm-season grass, seashore paspalum, owing to its higher biomass yields and tolerance to soil salinity. We observed that the plant heights and leaf–stem ratios were significantly affected by the ecotype of the plants. However, the height and leaf–stem ratio of seashore paspalum plants were not important factors in determining the yield and had no significant correlation with the FW or DW of the plants. There were no significant differences between the chlorophyll content and Pn of the majority of ecotypes studied herein. Consequently, the forage yield of seashore paspalum was more likely determined by the tillering ability than the genetic makeup of each ecotype. This finding differed from the results of some studies which demonstrated that the fresh and dry forage yields of alfalfa cultivars are mostly attributed to high leaf to shoot ratio, plant height, and chlorophyll content (Monirifar et al., 2020). The improvement of chlorophyll content and photosynthetic capacity is an important factor that influences the yield of alfalfa (Cornacchione and Suarez, 2017). Generally, leaf tissues have higher forage quality than stem tissues, and a marked increase in the leaf–stem ratio of forage grasses is expected to improve forage quality (Casler et al., 2014; Sanchez et al., 2018). A higher leaf–stem ratio significantly affects the CP content of rhizome peanut (*Arachis glabrata* Benth.) ecotypes (Alencar et al., 2019) and alfalfa (Hakl et al., 2016). However, the results of this study suggested that the leaf–stem ratio and the forage nutritive value, indicated by the CP content, EE content, NDF, and ADF, were not significantly correlated. This could be attributed to the low density and lignification of the stem in seashore paspalum.

### Effect of salinity on forage yield and CP content

Despite the excellent salt tolerance ability of seashore paspalum, the FW, DW, and DFR of the majority of ecotypes were influenced by high salinity. Salt stress significantly decreased the FW of most ecotypes. A previous study demonstrated that the dry mass of the shoot of two halophytic grasses, puccinellia (*Puccinellia ciliata* Bor. cv. Menemen) and tall wheatgrass (*Thinopyrum ponticum* Podp.), decreased by 50% following treatment with 300 mM NaCl, and both grasses barely survived under 600 mM NaCl (Jenkins et al., 2010). The growth of switchgrass (*Panicum virgatum* L.) EG 2101 has been shown to be severely affected by increasing levels of salinity, but it produces more biomass than populations of prairie cordgrass (*Partina pectinata* Link) under moderate salinity (5 dS m^−1^) (Anderson et al., 2015). In this study, high salinity (700 mM NaCl) decreased the FW and DW of seashore paspalum shoots by 50.6% and 23.6%, respectively, on average. The survival rate of the seashore paspalum ecotypes varied from 6.5% to 49.0% following treatment with 700 mM NaCl for 7 weeks.

The results of this study suggest that seashore paspalum has outstanding salt tolerance and forage quality at high salinity. As the growth of alfalfa and tall wheatgrass is stunted by 600 mM NaCl, their forage quality at high levels of salinity (above 300 mM NaCl) cannot be evaluated owing to insufficient sample size (Shahba et al., 2012; Sagers et al., 2017; Waldron et al., 2020). The CP content of seashore paspalum was found to be higher than that of Rhodes grass (*Chloris gayana* Kunth.) (11.5%) and Bermuda grass (*Cynodon dactylon* L. Pers.) (16%) but lower than that of alfalfa (22%), *Agropyron cristatum* (36%), and *Lolium perenne* (34%) (Pasternak et al., 1993; Lee, 2018). A previous study on 15 cultivars of wheat (*Triticum aestivum* L.) reported that the CP content varied from 20% to 24% (Kim and Anderson, 2015), while an oat (*Avena sativa* L.) cultivar (Forage Plus and Ogle) was reported to have a CP content of 13%–15% (Coblentz and Cavadini, 2016). The mean CP content of sporobolus grass (*Sporobolus virginicus*), a halophytic grass, was reported to be 8.7% of the dry matter, which was found to be higher than that of four other species of grass grown on the saline soils of Australia, with mean CP contents of 5%–8% of the dry matter. (Al-Shorepy et al., 2010; Norman et al., 2013). These findings indicate that there is no relationship between the CP content and soil salinity, and the effects of salinity on the CP content of grasses are inconsistent. The forage quality of the halophyte, forage kochia, is affected by the accumulation of salt when grown on saline soils containing nearly 300 mM of salt. A study demonstrated that the CP content of forage kochia following treatment with 600 mM NaCl decreased by 41% of that of the control (Waldron et al., 2020). The CP content of sporobolus grass was reported to increase from 6 to 9% when irrigated with increasingly saline water (Ashour et al., 1997). In this study, the CP content of seashore paspalum was not significantly affected at 700 mM NaCl. The CP content was 17.4% and 19.3% under control and saline (700 mM NaCl) conditions, respectively, with the exception of three ecotypes in which the CP content had increased significantly. The CP content of almost all the ecotypes of seashore paspalum was significantly higher than the recommended level for nursing calves (15% dry matter) and lactating cows (9%–12% dry matter) (Kim and Anderson, 2015). Furthermore, seashore paspalum can be an important source of antioxidants in saline environments. The results of this study are corroborated by those of several studies on various halophytes. Antioxidant enzymes play a key role in salt tolerance and improve plant growth by quenching reactive oxygen species and could therefore serve as a good source of protein for livestock (Tlili et al., 2020).

### Effect of salinity on fiber quality

The cell walls of forage plants contain large amounts of fiber that comprise NDF, ADF, and acid detergent lignin (ADL). NDF and ADF are very important indicators for determining the forage quality, and the contents of NDF and ADF are negatively correlated with forage digestibility by livestock (Surber et al., 2011; Xie et al., 2011; Sanz-Sáez et al., 2012). The NDF content is especially important when formulating feed rations for ruminant animals and correlates with forage intake (Buxton, 1996). High lignin content contributes to the low forage quality of some warm-season grasses (Giordano et al., 2014; Gitau et al., 2017). Statistical analyses have revealed that the mean values of NDF and ADF across all forage plants are 57% and 32%, respectively. The mean NDF and ADF content are highest in grasses, being 59% and 33%, respectively, and lowest in legumes, being 42% and 28%, respectively. Large variations in the values of NDF and ADF have been reported within species. The highest values of NDF measured in grasses are 39%–70% and 34%–62% in *Alopecurus pratensis* and *L*. *perenne*, respectively, while the highest values of ADF reported in grasses are 2%–35% and 18%–46% in *L*. *multiflorum* and *B*. *inermis*, respectively (Lee, 2018).

In this study, the ADF content was significantly correlated with the CP (*r* = 0.802, *P*<0.01) and EE (*r* = 0.548, *P*<0.05) contents under control conditions. This finding was different from the results of a study which reported that the ADF content is significantly negatively correlated with the CP content (de Araujo and Coulman, 2002). The average NDF and ADF contents of seashore paspalum were 57.4% and 29.8%, respectively, of the DW under control conditions, which were lower than those of the forage Bermuda grass. A study reported significant variations in the ADF (24.1% to 34.5%) and NDF (64.3% to 77.3%) contents of 168 plant introductions of forage Bermuda grass (Anderson et al., 2010). The ADF (20.6% to 24.3%) and NDF (39.4% to 46.5%) contents of wheat are markedly lower than those of seashore paspalum. The NDF content of old man saltbush (*Atriplex nummularia* L.) collected from diverse saline soils is also lower (30% to 45% of DW) than that of seashore paspalum. The ecotypes and salt stress had little influence on the RFV (91% to 115%) of seashore paspalum; however, it has been reported that the RFV of wheat (146% to 183%) is significantly affected by the variety. The superiority of the RFV is reflected in the lower values of ADF and NDF. However, higher values of RFV also result in the worst mean forage yield in some varieties of wheat (Kim and Anderson, 2015).

Forage plants undergo morphological and physiological changes that affect the NDF content and nutritive values (Arnold et al., 2019). However, the interactions between the salinity of the environment and the forage fiber value of grasses are not consistent (Norman et al., 2013). Our results suggest that short-term salt stress significantly affected the DW and reduced the NDF and ADF contents to a slight extent (50.2% and 25.9% of DW on average, respectively; *P >*0.05). The lower NDF content was beneficial for the higher dietary energy and improved livestock intake of seashore paspalum. It has been reported that the NDF contents of forage kochia are not affected by salinity (from 30% to 32.4%), while those of alfalfa and tall wheatgrass are only slightly affected by saline conditions (Waldron et al., 2020). Furthermore, a study demonstrated that there is no clear correlation between the NDF content and increasing salinity in river saltbush (Masters et al., 2010). These existing findings support the results of this study on seashore paspalum. The results of the present study further suggest that seashore paspalum has excellent salt tolerance ability and could also serve as a high-quality forage plant.

## Conclusions

Seashore paspalum is a halophytic warm-season grass with superior salt tolerance. Although the plant is primarily used in athletic fields and landscape areas as lawn grass, seashore paspalum is an attractive potential feedstock that is used for livestock feed (for hay and grazing) owing to its superior ground cover, biomass accumulation, and salt tolerance. The majority of studies on salt-tolerant forage plants emphasize elucidating the agronomic traits and underlying physiological mechanisms. Fewer studies have aimed to elucidate the effects of salinity on the forage quality of halophyte species. This study is the first to investigate the nutritive value of seashore paspalum and the influence of salinity on its nutritive value. Altogether, the results demonstrated that there were significant genetic (ecotype-specific) effects on plant heights and leaf–stem ratios in seashore paspalum. Salt stress significantly decreased the FW of the majority of ecotypes. However, seashore paspalum exhibited outstanding salt tolerance and forage quality at high salinity. Salt stress did not significantly affect the CP and EE contents of most ecotypes, but slightly reduced the NDF and ADF contents and improved the RFV. The results supported the fact that seashore paspalum is a good candidate for improving the forage base of saline soils. Seashore paspalum can serve as an excellent forage plant, providing high quality forage in saline soils. The study also focused on improving the productivity of saline systems by selecting ecotypes with higher nutritive value and biomass. For instance, the ecotype of 647907 selected in this study had the highest EE and CP content; however, the DW of the plants increased only slightly under high salt stress. At the same time, the interactions between the ecotypes and their environment should be considered when selecting ecotypes with higher feeding values. The cold and salt tolerance of the ecotypes was additionally evaluated herein, and the results could provide a basis for the selection of different ecotypes at different climates and salinities.

## Data availability statement

The raw data supporting the conclusions of this article will be made available by the authors, without undue reservation.

## Authors contributions

KJ conducted experiments. ZY, JS, HL, SC, YZ, WX, and WL give advice and assistance in this research. ZW revised manuscript. XW designed experiments and wrote manuscript. All authors contributed to the article and approved the submitted version.

## Funding

This work was supported by the National Natural Science Foundation of China (32101423), the Foundation Project of Shandong Natural Science Foundation (ZR2021MC066), the Fundamental Research Funds for the Universities (6631120002), and the ‘First Class Grassland Science Discipline’ programme of Shandong Province.

## Acknowledgments

The authors would like to acknowledge Meng Shi, Ying Chen, Rui Zhao, Xiaofan Wu, Wenjian Xu, and Dongyang Liu for their help on the article and also thank the National Foundation and the Key Laboratory of the Yellow River Delta Grassland Resources and Ecology of the Chinese Forestry and Grassland Administration for the support of this project.

## Conflict of interest

The authors declare that the research was conducted in the absence of any commercial or financial relationships that could be construed as a potential conflict of interest.

## Publisher’s note

All claims expressed in this article are solely those of the authors and do not necessarily represent those of their affiliated organizations, or those of the publisher, the editors and the reviewers. Any product that may be evaluated in this article, or claim that may be made by its manufacturer, is not guaranteed or endorsed by the publisher.

## Abbreviations

Pn, net photosynthetic rate; Ci, intercellular CO_2_ concentration; Tr, transpiration rate; Gs, stomatal conductance; FW, fresh weight; DFR, dry-fresh weight ratio; DW, dry weight; CP, crude protein; EE, ether extract; NDF, neutral detergent fiber; ADF, acid detergent fiber; DDM, digestible dry matter; DMI, dry-matter intake; RFV, relative feed value.
